# Arizona Registered Dietitians Show Gaps in Knowledge of Bean Health Benefits

**DOI:** 10.3390/nu10010052

**Published:** 2018-01-08

**Authors:** Donna M. Winham, Andrea M. Hutchins, Sharon V. Thompson, Mariah K. Dougherty

**Affiliations:** 1 Department of Food Science & Human Nutrition, Iowa State University, Ames, IA 50011, USA; 2 Department of Health Sciences, University of Colorado, Colorado Springs, CO 80918, USA; Andrea.Hutchins@uccs.edu; 3 Division of Nutritional Sciences, University of Illinois at Urbana Champaign, Urbana, IL 61801, USA; svthomps2@illinois.edu; 4 Department of Pharmaceutical & Nutrition Care, University of Nebraska Medical Center, Omaha, NE 68198, USA; mariahkay21@gmail.com

**Keywords:** legumes, pulses, attitude, chronic disease, consumption, nutrition, health professionals

## Abstract

Registered Dietitians (RDs) promote nutrition practices and policies and can influence food consumption patterns to include nutrient dense foods such as beans. Although many evidence-based health benefits of bean consumption (e.g., cholesterol reduction, glycemic control) have been demonstrated, there is limited research on the knowledge, attitudes, and perceptions of RDs regarding the inclusion of beans in a healthy diet. To fill this existing research gap, this cross-sectional survey explored the perceptions, knowledge, and attitudes of 296 RDs in Arizona, USA, toward beans. The RDs largely held positive attitudes toward the healthfulness of beans and were aware of many health benefits. Some gaps in awareness were evident, including effect on cancer risk, intestinal health benefits, folate content, and application with celiac disease patients. RDs with greater personal bean consumption had significantly higher bean health benefit knowledge. Twenty-nine percent of the RDs did not know the meaning of ‘legume’, and over two-thirds could not define the term ‘pulse’. It is essential that RDs have up-to-date, evidence-based information regarding bean benefits to provide appropriate education to patients, clients, and the public.

## 1. Introduction

Beans and other dry grain pulses such as peas, lentils, cowpeas, pigeon peas, and chickpeas represent a diversity of flavors, tastes, and textures, but all are nutrient-dense and beneficial for human health. Pulses are classified as the dry seeds of certain legume crops that are used as staple grains around the world. Whereas most vegetables are gathered when green, pulses are collected when dry. They are a subgroup of the legume plant family *Fabaceae* that also includes soybeans and peanuts. There has long been evidence for reductions in chronic disease risk with dry bean (*Phaseolus vulgaris* L.) consumption like pinto, black, kidney, white, and navy [1]. Epidemiological studies indicate dietary patterns high in beans are associated with greater longevity [2,3]. Specific health benefits of bean intake include lowering of blood lipids and reduced cardiovascular disease risk [4,5,6], improved postprandial glycemic control [7], and reductions in the risk of some cancers [8]. Beans and other pulses are high in dietary fiber and plant protein that can increase satiety [9,10], and reduce body weight [11].

Based on the evidence for the health benefits of pulse consumption, the US Dietary Guidelines for Americans Committee promoted them in the diet as both a vegetable and protein source in 2005 and 2010 [12,13]. The 2015 Dietary Guidelines for Americans (DGA) recommendation for the consumption of dry grain legumes is 1.5–2 cups per week for a 2000-calorie diet [14]. However, despite this guidance, routine bean or dry grain pulse consumption is achieved by a minority (8%) of the US population [1,15]. Higher pulse intakes have been observed among specific ethnic groups such as Hispanics, African Americans, and individuals of Asian Indian or Middle Eastern descent, but dietary acculturation and time constraints may result in decreased pulse consumption over time [1,15,16].

A Registered Dietitian (RD), or the equivalent term Registered Dietitian Nutritionist (RDN) implemented in 2014, is a food and nutrition expert who has met academic and professional training requirements. Over one-third of RDs work in clinical and outpatient settings, and provide counseling for the treatment and prevention of health conditions, such as type 2 diabetes and cardiovascular disease. RDs also provide nutrition counseling to numerous community members through federal nutrition programs. RDs are the front-line spokespeople for nutrition, function at the interface of medicine and public health, and translate evidence-based research into common language. In the US, about 76% of the approximately 97,000 RDs report their ethnicity as white, and 89% are women, but their clientele is gender and culturally diverse [17]. To reach the public successfully, RDs must have a high level of cultural competency, be able to inform about socially appropriate ways achieve optimal nutrition, and have an understanding of traditional foods [18]. These skills are particularly needed when it comes to promoting nutritional habits for persons with type 2 diabetes and other chronic diseases [19,20]. RDs may also precept interns, and thus their attitudes and breadth of experience influences the next generation of dietitians as well [21].

While the advantages of pulse consumption are well-documented in the scientific literature, there is a gap in knowledge regarding RDs’ awareness of the health benefits of beans, and their practices with respect to bean promotion to consumers or clients. To date, only one known study has been conducted to assess RD understanding or support of bean or legume consumption. This sample of 256 Canadian RDs reported frequent legume recommendations, and favorable perceptions, preferences, and knowledge of legumes. Personal consumption of legumes by study participants was positively correlated with their knowledge level [22]. While the Canadian survey provides valuable insight into RD practices, it did not incorporate questions regarding RD knowledge of pulse health benefits. There remains little to no data regarding pulse-related knowledge, perceptions, and counseling practices among US RDs who may be less likely to promote pulse consumption in a country where low intakes are more common than in Canada (8% vs. 13% daily consumption, respectively) [1,23]. Additionally, the body of evidence to support the inclusion of beans and other pulses in the diet has expanded since the Canadian study’s 2001 publication. An increase in nutritional knowledge and promotion of beans by RDs may theoretically help consumers to improve their own health.

The objectives of the present study were to: (1) assess RD knowledge about the role of beans in a healthy diet and for the prevention and treatment of chronic disease; (2) determine RD attitudes and perceptions about dry and canned bean consumption; (3) describe RD bean consumption patterns; and (4) evaluate knowledge of the terms ‘legume’ and ‘pulses’ among RDs. We hypothesized that RDs would be aware of the full range of health benefits of beans for the treatment of chronic diseases and health conditions. Goals 2, 3, and 4 were exploratory, and as such were not hypothesis-driven.

## 2. Materials and Methods

### 2.1. Study Design and Participants

This cross-sectional study targeted a convenience sample of RDs residing in Arizona, USA. Direct email invitations to take the survey went to 1416 individuals on the Arizona Dietetic Association listserv. The proportion of inactive or multiple emails for the same individual was unknown. Survey Monkey (Palo Alto, CA, USA) software was used to collect online responses between September and October of 2012. Email invitation subject lines did not specifically inquire about beans, but rather requested participant expertise on nutrition and the role of functional foods in chronic disease. Subject lines were written in this fashion to minimize selection bias from individuals with a more pronounced understanding of legumes. The online survey took approximately 10–15 min to complete. Two reminder emails were sent 7–10 days apart. This study was classified as exempt by the Arizona State University Institutional Review Board (#1110006958). Voluntary consent was obtained through survey completion. For every 50 respondents who entered a separate raffle link, a random name was drawn for a $50 gift card to a major retailer. Respondents who provided an address were mailed a packet of manufacturers’ coupons valued at $3.

### 2.2. Survey Development

The survey instrument was developed from a literature review and adaptation of questions from other surveys that explored similar legume issues with consumers and health professionals [22,24]. During formative research, five RDs who worked in community nutrition, clinical nutrition, and diabetes education were asked about incentives and obstacles associated with beans and client consumption barriers and motivators. These responses were integrated into the survey. A formal online pilot was conducted with 19 RDs. The final questionnaire was administered following construct and content validity feedback from pilot test responders. Formative research and pilot test participants were not included in the final analysis.

Factual knowledge topics were derived from existing scientific literature related to the nutrient characteristics, health benefits, and chronic disease risk reduction of beans. These questions were structured as 5-point Likert-type questions ranging from strongly disagree, disagree, agree, strongly agree, or do not know. Perceptions and attitudes about bean consumption, including both dry and canned beans, were drawn from previous research with consumers, and documented barriers to consumption [22,24]. This second group of Likert-type questions included the option of ‘neutral’ in place of ‘do not know’ as a response category. Participants answered demographic questions and queries about their employment status, workplace type, type and years of dietary counselling experience, personal bean consumption and cooking patterns, and knowledge of ‘legume’ and ‘pulse’ definitions.

### 2.3. Data Analysis and Variable Transformation

Descriptive statistics were examined for normality and distribution of variable responses. Bean consumption frequencies were collapsed from five into three categories due to small samples in the extremes of ‘once a month or less’ and ‘5+ times per week’. After examination of the frequency distribution for percent time spent counseling clients, this variable was recoded into two categories. One category included those who did not counsel at all or counseled less than 11% of their work time. The second category represented those RDs who counseled 11% or more of their time.

All reported statistical tests were two-sided with significance set at *p* < 0.05 using IBM SPSS Statistics for Windows, version 24 (IBM Corp., Armonk, NY, USA). Chi-square analysis was used to examine differences in Likert-type question responses for health benefits knowledge items, attitudes and perceptions towards bean consumption, and legume and pulse terminology. These comparisons were by the bivariate or trivariate variables: current client counselling responsibilities (yes/no), education level (Bachelors/Masters or higher), or personal bean consumption (≤2–3 times per month, 1–2 times per week, ≥3 times per week). The Likert-type questions were entered into the principal components analysis to identify thematic constructs. One factor emerged that represented the health benefits of beans. The resulting 6-item Likert scale was normally distributed and had a Cronbach’s alpha of 0.76, indicating good reliability.

## 3. Results

### 3.1. Response Rate

**Figure 1:**
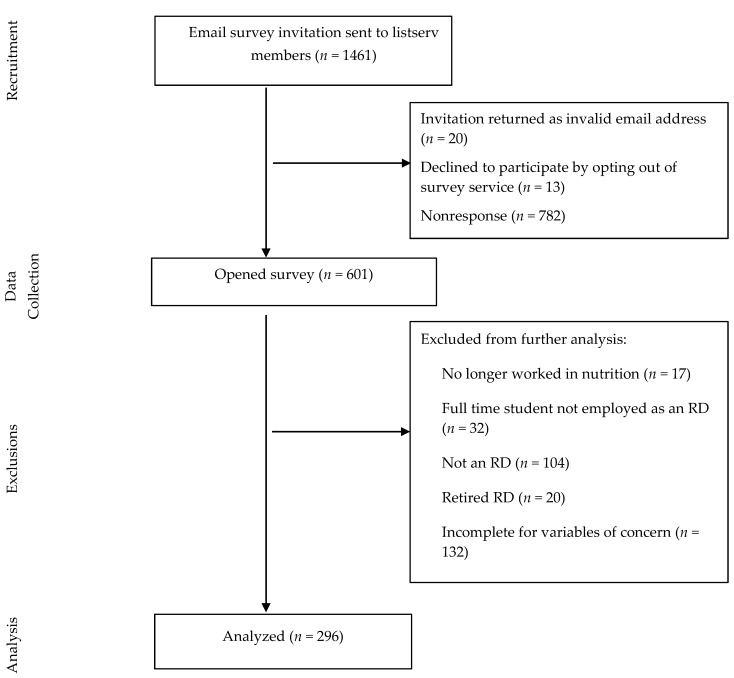
Consort flow diagram for participants of the Arizona Registered Dietitian Bean Health Benefits Survey.

Of the 1461 initial email invitations, 20 were invalid addresses and returned, and 13 RDs opted out of receiving further messages. Fifty-four percent (782) of the initial messages did not elicit a response. Of the 601 surveys opened (43%), some cases were excluded from further analysis if the respondent: no longer worked in nutrition (*n* = 17); was a full-time student not employed as a RD (*n* = 32); was not a RD (*n* = 104); reported being retired (*n* = 20); or the survey was incomplete for the variables of concern (*n* = 132). The completion rate for the 428 eligible RDs was 69% (296/428). Figure 1 shows the CONSORT diagram for the sample.

### 3.2. Respondent Demographics

Demographic characteristics of the 296 respondents are presented in Table 1. The majority of respondents were White (95.4%) and female (95.6%), characteristics, which are similar to national RD demographics [17,25]. About five percent (*n* = 15) of the convenience sample reported their race as non-White, and included Chinese, Japanese, Hawaiian, Asian Indian, African American, Native American, and multiracial individuals. The mean respondent age was 43.7 (±12.5) years old, with 56.2% holding a Master’s degree or higher, a value higher than the national average (48% master’s, 4% doctoral) [25]. Over 44% of the participants were employed in clinical nutrition, which included inpatient (24.2%), outpatient (15.4%), and long-term care (4.6%). The next most common employment categories were community nutrition (21.8%), education and research (15.8%), food and nutrition management (11.2%), and lastly, consultation and business at 7.0%. Most respondents personally ate beans (98.0%) and reported that they knew how to cook them (88.2%).

### 3.3. Knowledge of the Health Benefits and Nutrient Content of Beans

Participant responses for the health benefits and nutrient content of beans are shown in Table 2. RD knowledge of the role of beans in low-density lipoprotein (LDL) cholesterol reduction, satiety, overall nutrition improvement, lowering cancer risk, and controlling blood glucose was high [4,5,6,7,8,9,10,11,26,27]. However, some bean health benefits were not as well known or incorrect. Forty-one percent of respondents were not aware that eating beans could improve child growth [28,29]. About 24% did not know that bean consumption has been shown to increase the relative abundance of beneficial gastrointestinal bacteria [30,31,32]. About 13% were unaware that persons with celiac disease could eat beans [33]. Twenty-six percent erroneously agreed or strongly agreed that beans lowered HDL cholesterol and 11.8% did not know the answer [4]. About 52% of RDs believed that bean consumption could reduce constipation [34]. When asked if regular consumption of beans or eating beans on most days of the week can increase protein in the diet, almost 100% of respondents agreed or strongly agreed. While the protein content of beans was a well-known fact, other nutrition statements were less recognized. About 77% of RDs agreed that beans increased dietary folate, 16.5% did not know this fact, and 6.2% disagreed with this true statement. Fifty-one percent of respondents erroneously disagreed that interference with mineral absorption was possible due to bean phytochemicals, and over 30% did not know [35].

Chi-square analysis of eight of the 18 Likert-type statements on health benefits and nutrient characteristics of beans, showed significant differences by the categorical variables of bean consumption, counseling status, and education level. RDs who consumed beans more often agreed or strongly agreed that eating beans can improve your nutrition, lower cancer risk, increase good gastrointestinal bacteria, and decrease mineral absorption. They also disagreed that beans can lower HDL or decrease dietary fiber. RDs with lower bean consumption were more likely to state that beans caused bloating. RDs with a Bachelor’s degree were more likely to state ‘do not know’ for statements on phytochemical interference with mineral absorption and increase good gut bacteria. The 6-item ‘health benefits of beans’ scale had a mean of 3.35 ± 0.55 and ranged from 0.8–4. There were no significant differences in mean scale values by the categorical bean consumption frequency, counseling percentage, or education level. Multivariate regression modeling was not significant using gender, age, education, bean consumption frequency, and counseling frequency.

### 3.4. Attitudes and Perceptions of Bean Consumption and Preparation

Table 3 shows RD responses to survey questions on attitudes and perceptions regarding cooked dry beans and canned beans. Overall, individuals reported positive attitudes and perceptions toward the taste of beans, their preparation, and their consumption. The majority responded they liked the taste (92.2%) and the texture of beans (93.6%), and that beans were easy to use in meals (82.7%). Almost all of the RDs disagreed with the statement that ‘only poor people eat beans’ (97.6%). When looking at opinions of beans, the majority stated family, children, and friends would eat beans, while disagreeing that their family would only eat homemade, not canned, beans. Cultural or heritage perceptions about cooking dry beans varied among respondents. While 20.7% agreed that cooking dry beans was a part of their culture, more than half (62.7%) disagreed with this statement, and 16.6% remained neutral on the topic. A slight majority of the RDs disagreed that beans take too long to cook (42.2%), compared to those who agreed with this statement (34.1%). This left almost a quarter of the respondents in the neutral category for this bean cooking duration question. The majority of respondents (83.3%) disagreed or were neutral on the statement that bean consumption produces intestinal gas. Most respondents (54.3%) took a neutral stance on the statement about genetic modification of dry beans in the US at 54.3%. More people incorrectly agreed with this question than disagreed (35.1% vs. 10.6%). Genetically modified (GM) beans are not sold in the USA [36].

Similar results were observed between the general bean questions and canned bean questions. The majority of RDs disagreed that canned beans do not taste good (84.9%) or they did not like the texture of canned beans (88.2%). Most respondents agreed that canned beans were affordable (89.9%) and that they would eat canned beans (93.6%). About 89% of respondents disagreed that buying canned beans was not true to their culture or heritage, and agreed that their family would eat canned beans. Statements that received the highest neutral responses included ‘canned beans are as healthy as home-made beans’ at 26.8% and ‘canned beans have preservatives in them’ at 33.7%. While more respondents agreed that canned beans are as healthy as homemade beans (50.7%), the population was divided in thirds when queried on preservatives. Only 33.4% correctly disagreed that canned beans have preservatives, as the canning process is what preserves the food. A slight majority of RDs agreed that canned beans contain too much salt (41.9%), compared to those who disagreed (24.4%) and remained neutral (33.6%).

**Figure 2:**
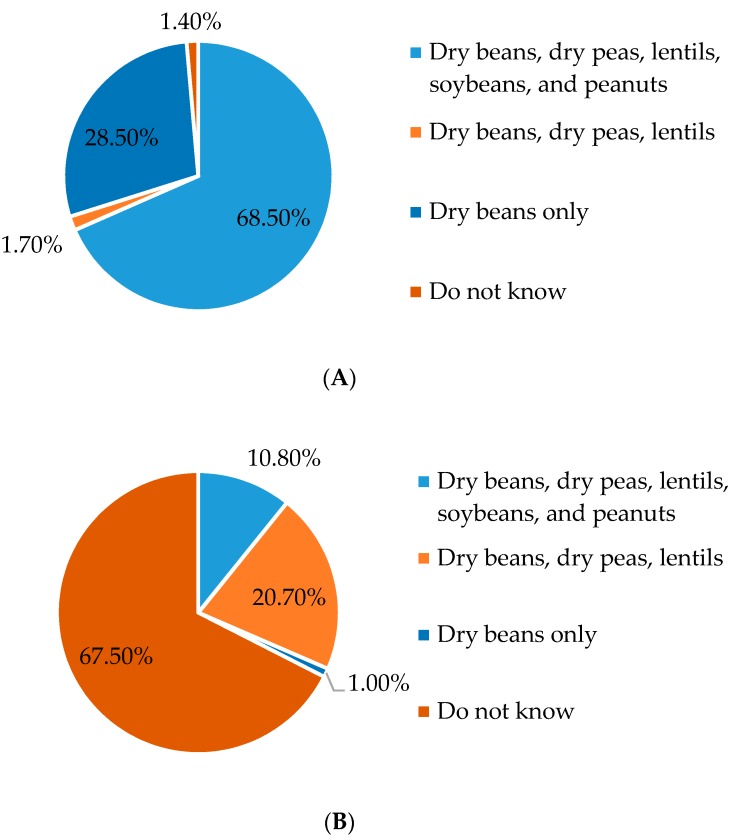
Response percentages for knowledge of the terms (**A**) ‘legumes’ and (**B**) ‘pulses’ by Arizona Registered Dietitians (*n* = 296).

While 68.5% of RDs knew the appropriate definition of a legume (dry beans, dry peas, lentils, soybeans, and peanuts), almost the same portion (67.5%) did not know the definition of pulses (Figure 2). About 21% answered the pulse question correctly (dry beans, peas, and lentils). Eleven percent thought the pulse and legume definitions were the same. Pulse definition responses were significantly different by education level. More RDs with an advanced degree correctly identified the term pulses (27.9% vs. 11.6%) than those with a Bachelor’s degree or less.

## 4. Discussion

Our primary study objective was to (1) assess knowledge about the role of beans such as pinto, black, kidney, or navy (*P. vulgaris* L.) in the prevention and treatment of chronic disease and general nutrition among Arizona RDs. We also sought to: (2) determine the attitudes and perceptions about dry and canned bean consumption; (3) describe bean consumption patterns; and (4) evaluate knowledge of the terms ‘legumes’ and ‘pulses’. Study results indicate that Arizona RDs had positive attitudes and perceptions about legumes for their own personal consumption and that of their clients. The majority of study participants displayed adequate knowledge regarding the health benefits of beans. 

However, there were some areas where a subset of RDs seemed deficient in understanding regarding the full range of bean health benefits. Approximately 9–26% of participants lacked knowledge of the key benefits of beans such as their high folate content, potential ability to lower cancer risk, and positive influence on intestinal health. Other issues of health-related importance to clients and consumers that were not fully understood by RDs were the role of beans in improving child growth and the acceptability of beans for those who have celiac disease. The respondents were equally split between those who thought beans improved child growth versus those who stated they did not know. This is a missed opportunity for RDs to promote nutrient and fiber-rich beans to children as not only a protein source, but also an easy to prepare vegetable. About 13% of nutrition professionals answered, “do not know” if beans were bad for persons with celiac disease. While only about 1% of the general US population has celiac disease [37], a high portion of these individuals will be referred to RDs for disease management. Lack of RD understanding of legume options within a gluten-free diet could be detrimental if dietary recommendations omit these highly nutritious foods to those with celiac disease [33,38]. 

Other identified misperceptions included genetic modification in dry beans and preservatives used in canned bean products. Beans and other pulses sold in the US are not genetically-modified [36]. Public concern about non-GM crops should be addressed with facts and recommendation of alternative non-GM foods such as pulses to concerned consumers [39]. Canned beans are preserved by processing and typically do not contain other chemical preservatives. Commercial canning processors have reduced sodium in canned beans in recent years due to consumer demands. For clients with sodium concerns, discarding the canning liquid and/or rinsing the product has been shown to reduce sodium content by 36–41% [40]. Canned foods offer shelf-stability, quick preparation time, and cost savings over fresh.

Bean consumption has been linked with reduced disease risk in previous studies [1,2,3,4,5,6,7,8]. Thus, increasing knowledge of the health benefits of beans may be a feasible approach to manifesting behavior change and reducing chronic disease risk. This is especially true in the case of the ‘fiber gap’ or low intakes of dietary fiber observed among most Americans. Despite decades of dietary advice to consume more fiber to reduce disease risk, 50–70% of adult Americans fail to meet the 28–42 grams per day recommendations [12,13,14,41].

Promotion of legumes for improving diet quality, increasing dietary fiber intakes, reducing cholesterol, and improving glycemic response are important health education messages for all populations, but especially those at added risk for nutritional deficits and for developing chronic diseases [42]. National consumption data [1,15], as well as more localized analyses of dietary patterns [16,24,43,44], suggests that bean consumption declines, while chronic disease risk increases, with greater acculturation to the Western diet [42,45]. Increasing awareness of these health benefits could help retain beans in the diets of limited resource groups, including immigrants. This information is particularly important for RDs in Arizona where cardiovascular disease, cancer, stroke, and diabetes rank in the top five causes of death across all ethnic groups [46]. Higher bean consumption has been associated with lower risk of these lifestyle-related diseases. 

While the majority of RDs could define the term legume, few were familiar with the term pulse in 2012. Since then there has been a global movement by domestic and international organizations to promote pulse crops, use the specific term ‘pulse’ to distinguish them from other legumes, and to standardize terminology. The United Nations Declaration of 2016 as the International Year of Pulses sparked a global movement by pulse organizations [47]. Extensive educational materials and outreach efforts were made to reach RDs and other health professionals in the US and beyond [47,48].

As RDs are experts who promote nutrition policy, their gaps in knowledge about health benefits of beans can affect federal policy, consumption patterns, and retention of bean intakes among immigrant and minority populations. In accordance with policy recommendations of the DGA for legume consumption, as well as the guidelines for dry pulses in WIC and SNAP nutrition assistance programs, it is essential that RDs be made more aware of the health benefits of beans. Efforts to encourage pulse education should come from federal nutrition and health agencies. These activities should also highlight ways to incorporate or maintain pulses in traditional diets in the face of dietary acculturation, urbanization, and an increasingly fast-paced food system [18,42,45]. 

There were some limitations to this study. The data were drawn from a convenience sample and only included RDs from Arizona, USA. Further research is needed to assess knowledge and any learning needs among RDs across the US to develop a generalizable picture of RD awareness of bean health benefits. Additionally, nearly all respondents stated that they consumed beans on a regular basis, which may have influenced responses. The email invitation asked about the “role of nutrition and chronic disease”, but survey completers may have been favorably biased toward beans. Overall, the survey questions focused on dry beans only, and did not ask about other pulses such as peas and lentils. 

While knowledge gaps existed, the survey identified many strengths, including positive attitudes about the overall healthfulness of beans and knowledge of health benefits such as lowering LDL cholesterol and improving iron and protein intake. Perceptions of canned beans continued to be positive and comparable to opinions of cooked beans. The majority of respondents liked the taste and attributes of canned and dry beans, and stated that they considered beans easy to prepare and appealing to family and friends.

## 5. Conclusions

Although Arizona RDs stated that they consume beans as a part of their own diets in the present study, they lacked knowledge about the full range of health benefits that beans provide for people affected by chronic disease, and thus may miss opportunities to emphasize the importance of legume consumption to their clients with these chronic diseases or conditions. Knowledge gaps regarding the health benefits of beans may further reduce consumption among the public served, resulting in an overall negative impact on consumer health. 

Nutrition education for disease prevention should promote bean consumption as part of a healthy lifestyle. Media outreach and education for lesser known aspects of bean nutrition should be identified and promoted among RDs. These findings aid in the development of nutrition-based messages to promote bean consumption in order to reduce chronic disease risk. Further research is needed to determine if the findings from this convenience sample of RDs are reflected in a larger random sample of RDs in the US. Translation of the evidence-based health benefits of beans to the public is mediated by the RD. It is imperative that RDs, as nutrition professionals, convey the important benefits of bean consumption and provide correct information to consumers.

## Figures and Tables

**Table 1:** Distribution of demographic, employment and personal bean practices of Arizona Registered Dietitians (*n* = 296).

Characteristics	Total
Age in years (±SD)	43.7 ± 12.5
Gender	
Female	95.6
Male	4.4
Hispanic ethnicity	
Yes	7.8
No	92.2
Self-reported race	
White	95.4
Other/multiracial	4.6
Education	
Bachelor’s degree	43.6
Master’s degree	49.8
Doctoral degree	6.4
Employment category ^a^	
Clinical nutrition	
Inpatient	24.2
Outpatient	15.4
Long term care	4.6
Community nutrition	21.8
Food & nutrition management	11.2
Consultation and business	7.0
Education	13.3
Research	2.5
Personal bean consumption	
Consumes beans	98.0
Does not consume beans	2.0
Bean consumption frequency	
Once a month or less	6.8
2–3 times per month	22.3
1–2 times per week	48.3
3–4 times per week	17.6
5+ times per week	5.1
Dry bean cooking knowledge	
Knows how	88.2
Does not know how	11.8
Bean cooking frequency	
Never	30.1
Once a month or less	43.6
2–3 times per month	19.3
1 time per week or more	7.1

^a^ *n* = 285.

**Table 2:** Percentage distribution of responses to statements of the health benefits of beans among Arizona Registered Dietitians (*n* = 296; T = true statement, F = false statement).

Eating Beans Can	Strongly Disagree	Disagree	Agree	Strongly Agree	Do Not Know
	%				
Help you feel full (T)	1.4	0.0	25.5	73.1	0.0
Improve your nutrition § ** (T)	1.4	0.0	33.4	64.5	0.7
Increase protein in diet (T)	0.0	1.0	42.9	55.8	0.3
Lower LDL cholesterol (T)	0.7	0.7	46.6	48.0	4.1
Reduce constipation (T)	0.0	3.8	51.5	43.7	1.0
Lower cancer risk § *; † * (T)	0.7	1.0	47.1	43.1	8.1
Control blood glucose † *(T)	0.3	4.4	52.5	38.3	4.4
Increase iron in the diet (T)	1.4	9.2	53.1	29.9	6.5
Increase dietary folate (T)	0.3	6.2	47.4	29.6	16.5
Help lose weight (T)	0.3	8.2	59.4	28.3	3.8
Increase ‘good’ bacteria in intestines § * (T)	0.7	7.8	45.1	22.2	24.2
Improve child growth (T)	1.0	6.7	37.5	13.0	41.8
Cause bloating § **; † * (T)	2.1	21.9	64.0	9.6	2.4
Decrease mineral absorption due to phytochemicals § ** (T)	12.3	38.9	15.2	1.4	31.7
Not suitable for persons with celiac disease (F)	44.1	39.7	3.1	0.3	12.9
Lower HDL cholesterol § ** (F)	32.1	30.4	13.2	12.5	11.8
Decrease fiber in the diet § * (F)	73.5	20.4	3.1	2.4	0.7

* *p* < 0.05; ** *p* < 0.01. § Significant for bean consumption categories; † Significant for counseling status.

**Table 3:** Perceptions about dry and canned beans among Arizona Registered Dietitians (*n* = 296).

Here Are Some Reasons People Have Said Prevent Them from Eating or Cooking Beans. … Do You …	Strongly Disagree	Disagree	Neutral	Agree	Strongly Agree
	%				
Like the taste of beans § **	1.4	1.7	4.8	41.5	50.7
Easy to make meals with beans § **	1.4	3.4	12.5	50.8	31.9
Canned beans are as healthy as home-made beans	2.7	19.8	26.8	37.9	12.8
Cooking dry beans part of culture	28.5	34.2	16.6	12.2	8.5
Some dry beans are genetically modified	3.1	7.5	54.3	26.6	8.5
Canned beans have too much salt in them	2.3	22.1	33.6	35.2	6.7
Dry beans take too long to prepare § *	11.6	30.6	23.8	29.3	4.8
Dislike the texture of beans § **	55.8	37.8	2.7	1.4	2.4
Canned beans have preservatives	8.1	25.3	33.7	31.0	2.0
Beans cause intestinal gas § *	12.2	34.7	36.4	15.0	1.7
Dislike the texture of canned beans	40.1	48.1	7.4	3.4	1.0
I will not eat canned beans	50.2	43.4	3.7	1.7	1.0
Friends do not eat beans	25.5	45.6	20.1	8.2	0.7
Canned beans do not taste good	37.2	47.7	12.1	2.3	0.7
Only poor people eat beans	73.2	24.4	1.7	0.0	0.7
Family will only eat home-made beans	35.6	46.4	13.2	4.4	0.3
Family will not eat canned beans	44.4	43.8	7.1	4.4	0.3
Family/children will not eat	47.1	38.6	8.8	5.4	0.0
Canned beans are too expensive	38.0	51.9	7.1	3.0	0.0
Canned beans not true to culture	51.7	37.6	9.1	1.7	0.0

§ Significant for bean frequency of consumption categories; * *p* < 0.05; ** *p* < 0.001.
